# The IL-17A modulating astrocytic activity was associated with the electroacupuncture-mediated improvement of sensorimotor ability after stroke

**DOI:** 10.1186/s13020-026-01446-5

**Published:** 2026-06-29

**Authors:** Yuenming Yau, Zeli Li, Zhiyuan Jiang, Jianyu Luo, Zhennan Wu, Chang Liu, Nenggui Xu, Lulu Yao

**Affiliations:** 1 South China Research Center for Acupuncture and Moxibustion, Guangzhou University of Chinese Medicine, Guangzhou, GD China; 2 Dongguan Hospital of Guangzhou University of Chinese Medicine, Dongguan Traditional Chinese Medicine Hospital, Dongguan, GD China; 3 Health Promotion Center for Primary and Secondary Schools of Guangzhou Municipality, Guangzhou, GD China

**Keywords:** Electroacupuncture, Two-photon calcium imaging, Astrocyte, Ischemic stroke, IL-17A

## Abstract

Both clinical and preclinical evidence showed that electroacupuncture (EA) at Baihui (Governing Vessel 20, GV20) and Dazhui (GV14) could relieve sensorimotor disability after ischemic stroke (IS). However, the mechanism behind this therapeutic remains to be unraveled, and the current findings of its underlying mechanism are mainly focusing on neurons, with little evidence from astrocyte. Astrocyte plays an essential role in IS pathology, and IL-17A as a critical mediator in IS. This study aimed to investigate the relationship between the activity of astrocyte and expression of IL-17A during the regulation of sensorimotor function. The sensorimotor-related behaviors in mice were assessed by using Adhesive removal test and Rotarod test, and the expression/function of IL-17A was evaluated by ELISA, RT-PCR, and Western Blot. In vivo calcium activity of astrocyte was detected by two-photon calcium imaging. Our results revealed that EA preconditioning promoted the recovery of sensorimotor function in IS mice, and this therapeutic effect was absent when the astrocytic activity was chemogenetically inhibited. In addition, astrocytic calcium activity of IS mice could be mimicked by overexpressing IL-17A. Altogether, these results suggested the IL-17A in the astrocyte may play a key role in the functional recovery after stroke. These findings not only elucidate a critical pathway for understanding how IL-17A in astrocytic calcium activity influences the functional recovery after stroke, but also suggest a potential valuable therapeutic strategy for stroke.

**Graphical Abstract:**

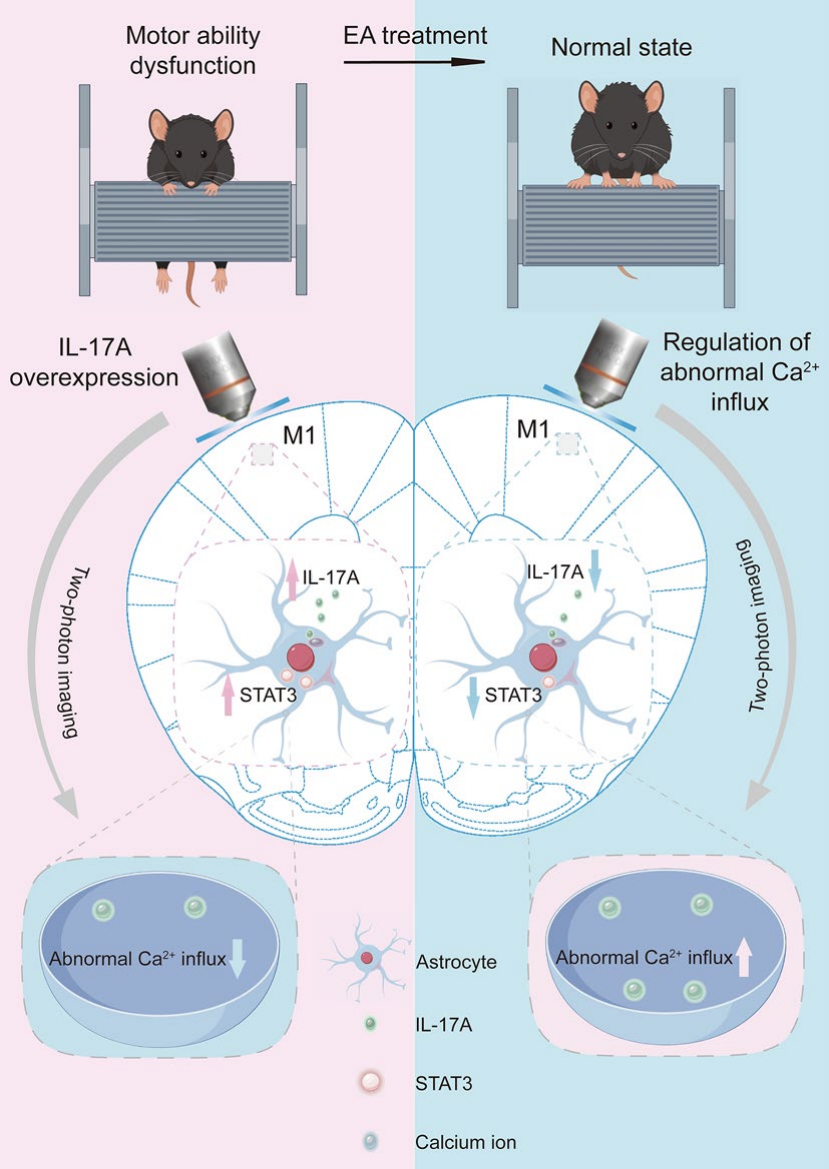

**Supplementary Information:**

The online version contains supplementary material available at 10.1186/s13020-026-01446-5.

## Introduction

Ischemic stroke (IS) is the third leading cause of death around the world according to the GBD 2021 Study [1, 2]. Despite advances in acute stroke management, effective long-term therapies to restore neurological function during recovery remain limited.

EA, a modern adaptation of acupuncture that combines electrical stimulation with traditional needle insertion, was traditionally used in East Asian medicine to rehabilitate, and treat chronic diseases and pain disorders [3, 4]. EA at GV20 and GV14 has been evidenced to be effective to the stroke functional recovery in both clinical and preclinical studies [5–7]. EA can suppress pro-inflammatory cytokines like IL-6 and TNF-α, thereby reducing systemic inflammation and promoting tissue repair, particularly in IS [8, 9]. Astrocyte-driven neuroinflammatory cascades critically contribute to IS pathogenesis [10]. However, the specific mechanisms by which EA influences astrocytic activity in the context of IS remain poorly understood.

Astrocytes could interact with neurons and regulate the inflammatory process in the stroke pathology [11–13]. Astrocytes drive context-dependent neuroinflammation through cytokine/chemokine secretion (IL-6, CXCL1) and matrix metalloproteinase-9 production [14, 15]. IL-17A has been demonstrated to influence a range of astrocytic activities, including AMPK/SREBPs and NF-κB/MAPK signaling pathways [16–20], and there is a potential bridge between calcium dynamics and STAT3 activation, which acts as a transcription factor that enhances astrocytic endoplasmic reticulum calcium release and signaling activity by upregulating channels to mediate central nervous system inflammation and repair [21]. A recent study reported that EA can downregulate IL-17A and suppress neurotoxicity-induced astrocyte activation in the acute phase of stroke [22], while it remains unknown whether and how IL-17A engages astrocytes at the level of calcium signaling, and whether this astrocytic activity contributed to EA's benefit during the recovery phase. Therefore, we focused on the role of IL-17A in the astrocytic activity during the EA-mediated improvement of stroke recovery in this study.

To resolve these questions, we established a mouse model induced by focal photothrombotic ischemic stroke to test the hypothesis that EA improves sensorimotor recovery by modulating IL-17A and regulating abnormal calcium activity in astrocytes.

## Materials and methods

### Animals and virus injection

Six-week-old male C57BL/6 mice (23–25 g; Guangdong Zhiyuan Biomedical Technology Co., Ltd., Guangzhou, China) were housed under standard conditions (24 ± 2 °C, 12 h light/dark cycle) with ad libitum access to food and water for at least 7 days prior to experimentation. All experimental procedures were approved by the Animal Experiment Center of Guangzhou University of Chinese Medicine (Ethics Approval No. 20240802004).

AAV2/5 and AAV2/9 serotype viral vectors were used for in vivo delivery in C57BL/6 mice throughout the study. The following recombinant adeno-associated viruses (rAAVs) were employed: rAAV2/5-GfaABC1D-GCaMP6f-WPRE-hGH polyA, rAAV2/5-GfaABC1D-mCherry-WPRE-SV40 polyA, rAAV2/5-GfaABC1D-hM4D (Gi)-mCherry-WPRE-SV40 polyA, rAAV2/5-GfaABC1D-jRGECO1a-WPREs, and rAAV2/9-GfaABC1D-IL-17A-P2A-EGFP-WPRE-SV40 polyA (all vectors were obtained from BrainVTA Co., Ltd., Wuhan, China). For calcium-imaging viruses, rAAV-GfaABC1D-GCaMP6f, and chemogenetic viruses, rAAV-GfaABC1D-hM4D(Gi)-mCherry, terminal immunofluorescence characterization validated expression distribution and astrocytic specificity. For overexpression viruses (rAAV-GfaABC1D-IL-17A-EGFP), two-photon imaging-derived EGFP visualization confirmed expression scope and cellular specificity. CNO administration protocol: intraperitoneal injection, 1 mg/kg dose (vehicle DMSO), administered 30 min prior to behavioral testing or imaging experiments. For prolonged experimental protocols, supplemental injections were administered every 24 h per standard practice.

### Photothrombotic ischemia model

This procedure was performed as previously described [23], with minor modifications. Briefly, photothrombotic ischemia was induced using Rose Bengal, a photosensitive dye that absorbs 530 nm wavelength light, producing reactive oxygen species and promoting localized thrombus formation, leading to focal cerebral ischemia and hypoxia. Mice were intraperitoneally injected with 0.2 mL of 1% Rose Bengal solution and anesthetized with 175 mg/kg of Avertin. After confirming anesthesia, the mice were placed in a stereotaxic apparatus. The scalp was shaved, disinfected, and a midline incision was made to expose the skull. Connective tissues were carefully removed to visualize the bregma, and the right primary motor cortex (M1) was identified and marked.

To prevent corneal damage during irradiation, erythromycin ophthalmic ointment was applied to both eyes. A 532 nm LMGL532-50mW fiber-coupled laser (pre-warmed prior to use) was directed at the marked cortical area through a perforated light shield, and the region was illuminated for 6 min at an intensity of 0.55 mW/mm^2^.

Following photothrombosis, the incision was sutured and disinfected. Mice were then placed on a thermostatically controlled heating pad until full recovery before being returned to their home cages.

### Acupuncture preconditioning

**Fig. 1:**
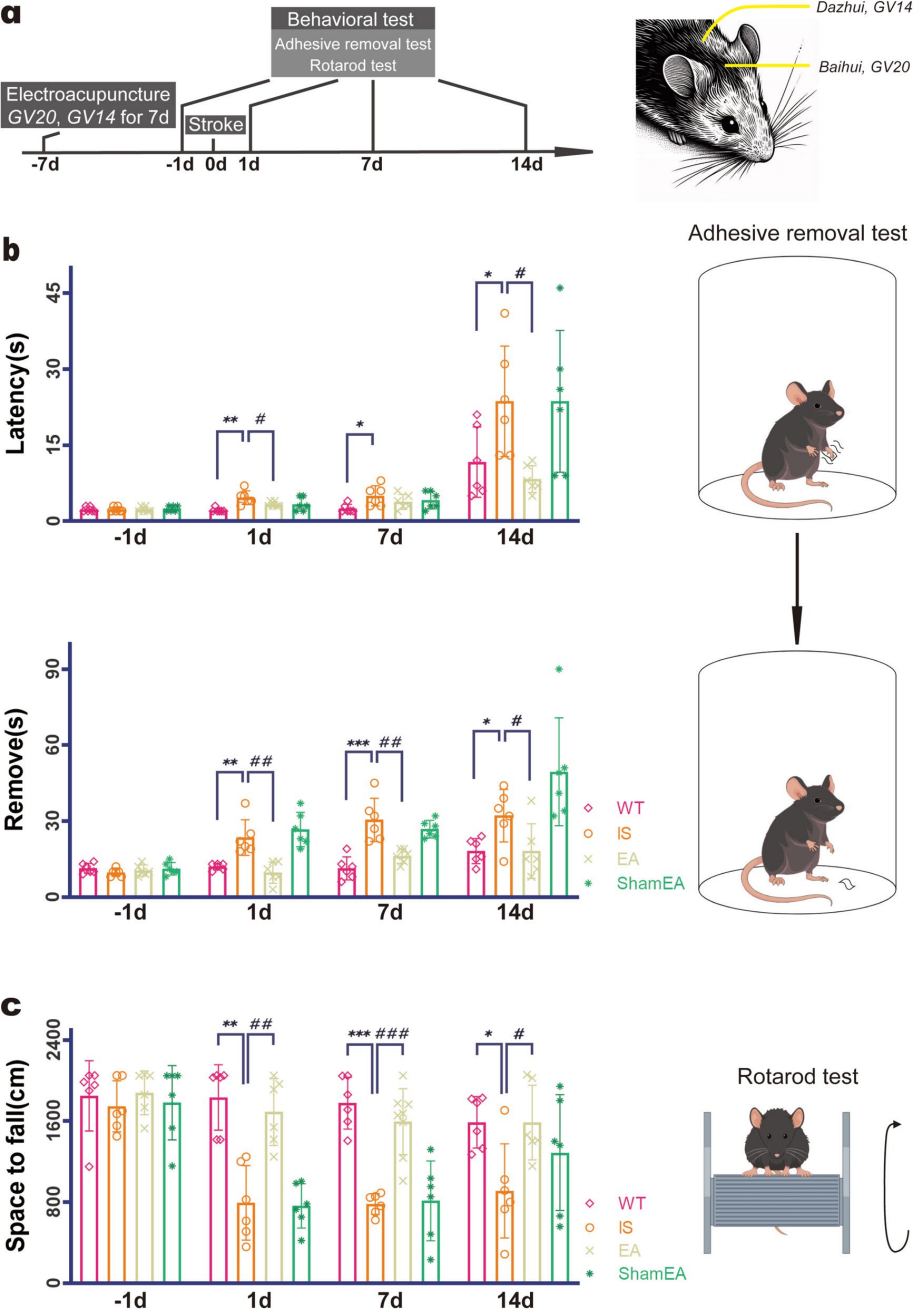
Electroacupuncture ameliorates motor disability in PTI-IS mice. **a** Right: Schematic of acupoints GV20 and GV14 in mice. Left: Experimental timeline. **b** Upper right: Schematic representation of mice detecting adhesive paper in the adhesive removal test, detection of sensory function in the left forelimb of mice. Upper left: Adhesive removal test results for pre- and post-IS on days 1, 7, and 14, latency to detect the sticky paper. Lower right: Schematic representation of a mouse tearing off an adhesive paper in the adhesive removal test, detection of the motor function of the left forelimb of the mouse. Lower left: Adhesive removal test results for pre- and post-IS on days 1, 7, and 14, time taken to remove the sticky paper (WT, IS, EA, ShamEA, n = 6). **c** Right: Schematic of the Rotarod test, detection of integral motor disability in mice. Left: Rotarod test results for pre- and post-IS on days 1, 7, and 14, total distance traveled in the Rotarod test (WT, IS, EA, ShamEA, n = 6). Results are expressed as the mean ± SD, *p < 0.05, **p < 0.01, ***p < 0.001 vs. WT; #*p* < 0.05, ## *p* < 0.01, ###*p* < 0.001 vs. IS. Effect size: Mann–Whitney test, 0.66 < r ≤ 1.0; Welch *t* test, 1.93 < Cohen’s d ≤ 2.26; *t* test, 0.10 < Cohen’s d ≤ 2.79

Mice were briefly anesthetized with isoflurane prior to electroacupuncture (EA) preconditioning. Stainless steel acupuncture needles (Suzhou Medical Supplies Factory Co., Ltd., Suzhou, China) were bilaterally inserted at acupoints [24] GV20 (Baihui) and GV14 (Dazhui). EA stimulation was delivered using a HANS-200A electroacupuncture apparatus (Han Institute, Indiana, USA) with continuous wave stimulation at a frequency of 2 Hz. The parameters used in this study were selected based on established preclinical literature and have demonstrated efficacy in both animal models and clinical trials for stroke rehabilitation [25, 26]. In clinical practice, EA at 2 Hz frequency is particularly relevant as it is associated with the release of endorphins and other neuropeptides that promote long-term neuromodulation and are considered safe and well-tolerated in humans [25]. The anatomical locations of the selected acupoints are illustrated in Fig. 1a. Acupuncture needles (Suzhou Medical Supplies Factory Co., Ltd., Suzhou, China) were inserted bilaterally at acupoints GV20 and GV14 to a depth of 3 mm.

### Behavioral tests

Behavioral assessments were conducted on days 1, 7, and 14 following IS, as well as one day prior to IS induction to obtain baseline measurements. The testing schedule is illustrated in Figs. 1a and 4a.

#### Adhesive removal test

Sensorimotor function was evaluated using the adhesive removal test. Briefly, mice were placed in a transparent cylindrical chamber (radius ~ 12 cm) and allowed to acclimate for 5 min. A 2 × 5 mm adhesive tape was affixed to the palm of the left forepaw. Mice were then placed into an identical transparent chamber for testing. The timer started when the mouse contacted the cage floor, and the latency to detect and remove the adhesive tape was recorded. After removal, the mouse was returned to its home cage. All trials were video recorded from two angles for subsequent analysis.

Before inducing stroke, all the mice underwent a standardized adaptation training for three consecutive days until their performance stabilized, thus establishing their individual baseline values. This ensured their familiarity with the task and minimized the differences among the groups caused by the learning process. The test was conducted in a constant environment (a soundproof behavior laboratory, during a fixed period of time). The test parameters included the size, brand, placement location (on the left front paw), application technique, and applied force of the tape. All of these were standardized and operated uniformly by a trained researcher, and were supervised by another researcher. All testing sessions were recorded from dual angles and analyzed by blinded raters, documenting both detection latencies and removal time, thereby eliminating observer bias. The testing chamber was cleaned with 75% ethanol between subjects to eliminate olfactory cues from preceding animals. Baseline data were collected one day before IS induction, and post-stroke performance was assessed on days 1, 7, and 14 after IS.

#### Rotarod test

Motor coordination and balance were assessed using the YLS-4D Rotarod Apparatus (Jinan Yanyi Technology Development Co., Ltd., Jinan, China). Mice randomized across groups to preclude weight-related confounding. We standardized as follows: all mice received three days of acclimation training pre-testing to eliminate neophobia and fear-based confounding. Testing occurred in quiet, dimmed environments under constant acceleration protocols (0–40 rpm/300 s), providing uniform, gentle stimulation. Each animal underwent three trials per session, with ≥ 30 min inter-trial intervals to preclude fatigue effects. During testing, mice were gently placed on the rotating rod by the tail, ensuring that both forelimbs and hindlimbs rested on the rod and that the animals back faced the direction of rotation. The latency to fall was recorded, and the total running distance was measured for each trial.

### Enzyme-linked immunosorbent assay (ELISA)

Cerebral tissue samples were retrieved from − 80 °C storage and thawed on ice. A total of 10 mg of each sample was weighed and homogenized in 10 μL of phosphate-buffered saline (PBS). The homogenates were centrifuged at 12,000 rpm for 20 min at 4 ℃, and the resulting supernatants were collected for analysis.

ELISA kits (Jiangsu Meimian Industry Co., Ltd., Yancheng, China) were used to quantify target proteins according to the manufacturer’s instructions. Blank, standard, and sample wells were prepared in parallel, with all samples run in technical replicates. After sealing the plate, it was incubated at 37 °C for 60 min, followed by five washing steps. Subsequently, chromogenic substrate was added, and plates were incubated for an additional 15 min at 37 °C in the dark. Optical density (OD) was measured at 450 nm within 15 min of the final incubation step. The concentrations of the analytes were calculated based on the standard curve.

### Transcriptomic analysis

Functional genomic data were retrieved from the Gene Expression Omnibus (GEO) database (https://www.ncbi.nlm.nih.gov/geo/). Using the keyword “stroke,” a total of 2917 datasets were identified, including 739 from Mus musculus. Among these, dataset GSE60820 and GSE163654, were included for comparative analysis. GSE60820 comprises gene expression profiles from five IS and five healthy mice, while GSE163654 includes data from six IS and six healthy mice.

Differentially expressed genes (DEGs) were identified using bioinformatics approaches. Raw data were normalized using *R* software (version 4.1.2), and quality control was visualized via volcano plots and heatmaps. DEGs were screened using the limma package with cut-off criteria of |log_2_ fold change| > 1 and *p* < 0.05. Common DEGs across datasets were determined using the Venny 2.1 platform to highlight genes enriched in the infarct core and peri-infarct (penumbra) regions.

A protein–protein interaction (PPI) network of DEGs was constructed using the STRING database, and hub genes were identified using Cytoscape software (version 3.9.1). Functional enrichment analysis, including Gene Ontology (GO) and Kyoto Encyclopedia of Genes and Genomes (KEGG) pathway analysis, was performed using the DAVID platform (*p* ≤ 0.05). GO analysis included biological processes (BP), molecular functions (MF), and cellular components (CC), while KEGG analysis elucidated key signaling pathways associated with stroke pathology.

It should be noted that there are differences in experimental conditions between our study and the bioinformatics analysis dataset. Our main objective is to investigate various stroke patterns, and different time windows to identify the ubiquitous and significantly dysregulated signaling pathways in ischemic central nervous system damage. Specifically, compared to GSE60820, their experimental subjects were mice in the acute stroke model; compared to GSE163654, their experimental subjects were rats and mice in the acute stroke model of pMCAO. Based on the comparative transcriptomic findings between the healthy and ischemic primary M1 regions, we determined that the healthy-side M1 region may serve as a more stable and informative target for downstream analyses. Therefore, all subsequent experiments in this study were conducted using the M1 region on the non-lesioned (healthy) hemisphere.

### Immunofluorescence

For brain tissue cryosectioning, mice were anesthetized via intraperitoneal injection of Avertin and transcardially perfused with phosphate-buffered saline (PBS), followed by 4% paraformaldehyde (PFA). Brains were post-fixed in 4% PFA at 4 °C for 24 h and then cryoprotected in 30% sucrose solution at 4 °C until fully saturated. Tissues were embedded in optimal cutting temperature (OCT) compound, snap-frozen in liquid nitrogen, and stored at − 20 °C. Coronal brain sections were prepared using a cryostat at a thickness of 40 μm.

For immunofluorescence staining, sections were rinsed in PBS three times for 10 min each, then incubated with blocking buffer (typically 5% normal serum and 0.3% Triton X-100 in PBS) for 1 h at room temperature. Sections were incubated overnight at 4 °C with the following primary antibodies: anti-STAT3 monoclonal antibody (MA5-15,739, Thermo Fisher Scientific, Waltham, MA, USA; 1:200 dilution) and anti-IL-17A antibody (ab79056, Abcam Inc., Cambridge, UK; 1:500 dilution). After washing, appropriate fluorophore-conjugated secondary antibodies were applied for 1 h at room temperature in the dark.

Nuclei were counterstained with 4',6-diamidino-2-phenylindole (DAPI, 1 μg/mL in PBS) for 10 min at room temperature. Sections were then rinsed, mounted with an anti-fade fluorescence mounting medium, and sealed.

Sections were then mounted using an anti-fade mounting medium and visualized using a laser scanning confocal microscope. Imaging was performed using a laser scanning confocal microscope (Nikon A1R-MP system, Nikon Inc., Tokyo, Japan). Image acquisition parameters were kept constant across groups. Quantitative fluorescence intensity and colocalization analysis were conducted using ImageJ software (National Institutes of Health, USA).

### Two-photon calcium imaging

#### Chronic cranial window implantation

To facilitate in vivo two-photon calcium imaging, a chronic cranial window was implanted in anesthetized mice. After scalp incision and full exposure of the skull, mice were positioned in a stereotaxic apparatus. A circular cranial window (6 mm in diameter) was carefully drilled using a 0.5 mm drill bit, centered at anterior–posterior (AP) = 0.98 mm and mediolateral (ML) = 1.8 mm, targeting the right M1.

The exposed area was immediately covered with a sterile circular glass coverslip, which was secured using photosensitive adhesive and cured with a dental light-curing system. A custom-designed stabilizing ring, compatible with the treadmill platform used for two-photon imaging, was fixed onto the skull using Z350XT Flowable Restorative (3 M Inc., USA) to ensure mechanical stability during imaging.

After surgery, mice were placed on a thermostatically controlled heating pad for recovery and monitored closely. A 14-day recovery period was provided to ensure sufficient wound healing and optical clarity. During the second postoperative week, mice were gradually acclimated to head-fixation and treadmill locomotion over several daily sessions to reduce stress and motion artifacts during subsequent imaging.

#### Two-photon imaging parameters

Two-photon calcium imaging was conducted using two independent imaging systems (Nikon and Leica). Mice were head-fixed on a custom-designed treadmill and allowed to acclimate for 30 min in a quiet, dimly lit environment. The cranial window was aligned under the objective lens, and a mode-locked Ti:Sapphire laser (Chameleon Vision II, Coherent Inc., USA) was used for excitation of either GCaMP6f or jRGECO1a. The focal plane was adjusted to a depth of 150–250 μm beneath the dura mater to visualize calcium transients in astrocytes within the M1 during spontaneous network activity.

For the Nikon A1R-MP system, imaging was performed using a 25 × water immersion objective lens, with a resolution of 512 × 512 pixels and laser power ranging from 10 to 28 mW. Data acquisition was carried out using NIS-Elements AR 4.60 software (Nikon Inc., Tokyo, Japan).

For the Leica STELLARIS 8 DIVE system, the same 25 × water immersion objective lens was used, with an image resolution of 1024 × 1024 pixels and laser power similarly set between 10 and 28 mW. Data were acquired using STELLARIS 8 DIVE software (Leica Microsystems, Heidelberg, Germany).

All imaging was performed on day 14 after IS induction.

#### Two-photon imaging data processing

Two-photon calcium imaging data were processed using NIS-Elements AR 4.60 software (Nikon Inc., Tokyo, Japan). Binary segmentation of imaging sequences was performed, and regions of interest (ROIs) corresponding to astrocytes were delineated using the software’s auto-thresholding function. Fluorescence intensity (F) was extracted for each frame over the entire recording period.

The baseline fluorescence (F₀) was defined as the average fluorescence during the lowest 5% of the signal, approximating one-twentieth of the total recording duration, and adjusted based on noise levels. ΔF/F_o_ was calculated as a normalized measure of fluorescence change:

$$\Delta {\mathrm{F}}/{\mathrm{F}}_{{0}} = \left( {{\mathrm{F}} - {\mathrm{F}}_{{0}} } \right)/{\mathrm{F}}_{{0}} .$$

The Total Ca^2^⁺ Integrated value was computed as the area under the ΔF/F₀ curve for each astrocytic ROI, reflecting the cumulative calcium activity over time. Calcium transients were defined as fluorescence signals exceeding a threshold set at mean + 2 × standard deviation of baseline noise. The frequency of calcium events was quantified as the number of transients crossing this threshold in 10 min.

### Statistical analysis

All statistical analyses were performed using GraphPad Prism software (version 8.0; GraphPad Software Inc., San Diego, CA, USA). Data from behavioral tests, ELISA, RT-PCR, Western blot, and two-photon calcium imaging were analyzed.

Normality of data distribution was assessed using the Shapiro–Wilk test with a significance level of α = 0.05. For data with normal distribution (*p* > 0.05), parametric tests were applied. For non-normally distributed data (*p* < 0.05), non-parametric tests such as the Mann–Whitney U test were used to avoid bias from inappropriate parametric assumptions.

For multiple group comparisons, one-way analysis of variance (ANOVA) was used, followed by pairwise comparisons with unpaired t-tests. Homogeneity of variance was assessed using the F test; if variances were unequal, Welch’s correction was applied to the t-test. *p* < 0.05 was considered statistically significant for all analyses.

## Results

### EA improved sensorimotor disability in the IS model by focal photothrombosis

A mouse model of unilateral IS with sensorimotor disability of the forelimb was established by performing photothrombotic surgery the right M1 area (M1FL) (AP: 0.98 mm, ML: 1.8 mm) [27]. EA intervention was performed 7 days before IS, and behavioral assessment was conducted before stroke as the baseline. Then, behavioral tests were carried out at 1, 7, and 14 days after stroke (Fig. 1a). On the 1st, 7th, and 14th day of the behavioral experiments to evaluate sensorimotor function, including the Adhesive removal test and Rotarod test. The results showed that the latency of detecting adhesive paper and the time of removing adhesive paper significantly increased in stroke mice, and the total distance of rotating rod was significantly reduced (Fig. 1b, c). Additionally, no upward trend was observed in other tests, with the exception of the adhesive paper detection latency, suggesting a lack of spontaneous recovery post-stroke.

Simultaneously, EA intervention demonstrated to be beneficial in sensorimotor recovery, with notable improvements in sensorimotor deficit behavior observed on post-stroke days 1 and 14. EA preconditioning can significantly reduce the latency of detecting adhesive paper in stroke mice, on the 1st, 7th and 14th day. Furthermore, the EA group exhibited a significantly shortened adhesive removal durations compared to controls. In the Rotarod test, pre-EA intervention on the days 1,7, and 14 after IS resulted in an increase in walking distance on the rotating rod (Fig. 1b, c). These results suggest EA preconditioning improved sensorimotor-related function after stroke, including adhesive paper detecting time, adhesive paper removal time, and enhanced sensorimotor capacity (Fig. 1b, c). Therefore, pre-intervention with EA stimulation can promote the sensorimotor functional recovery after stroke.

### Transcriptional profiling and KEGG pathway analysis identify IL-17A–associated differentially expressed genes in the IS model

To investigate potential key regulatory factors in IS, comparative transcriptomic analyses were performed on the M1 from IS model mice and healthy mice. Two datasets from the GEO database were utilized: GSE60820, comprising gene expression data from five IS and five healthy mice, and GSE163654, including six IS and six healthy controls. Differentially expressed genes (DEGs) between the IS-affected and contralateral (healthy) M1 regions were identified using *R* software.

**Fig. 2:**
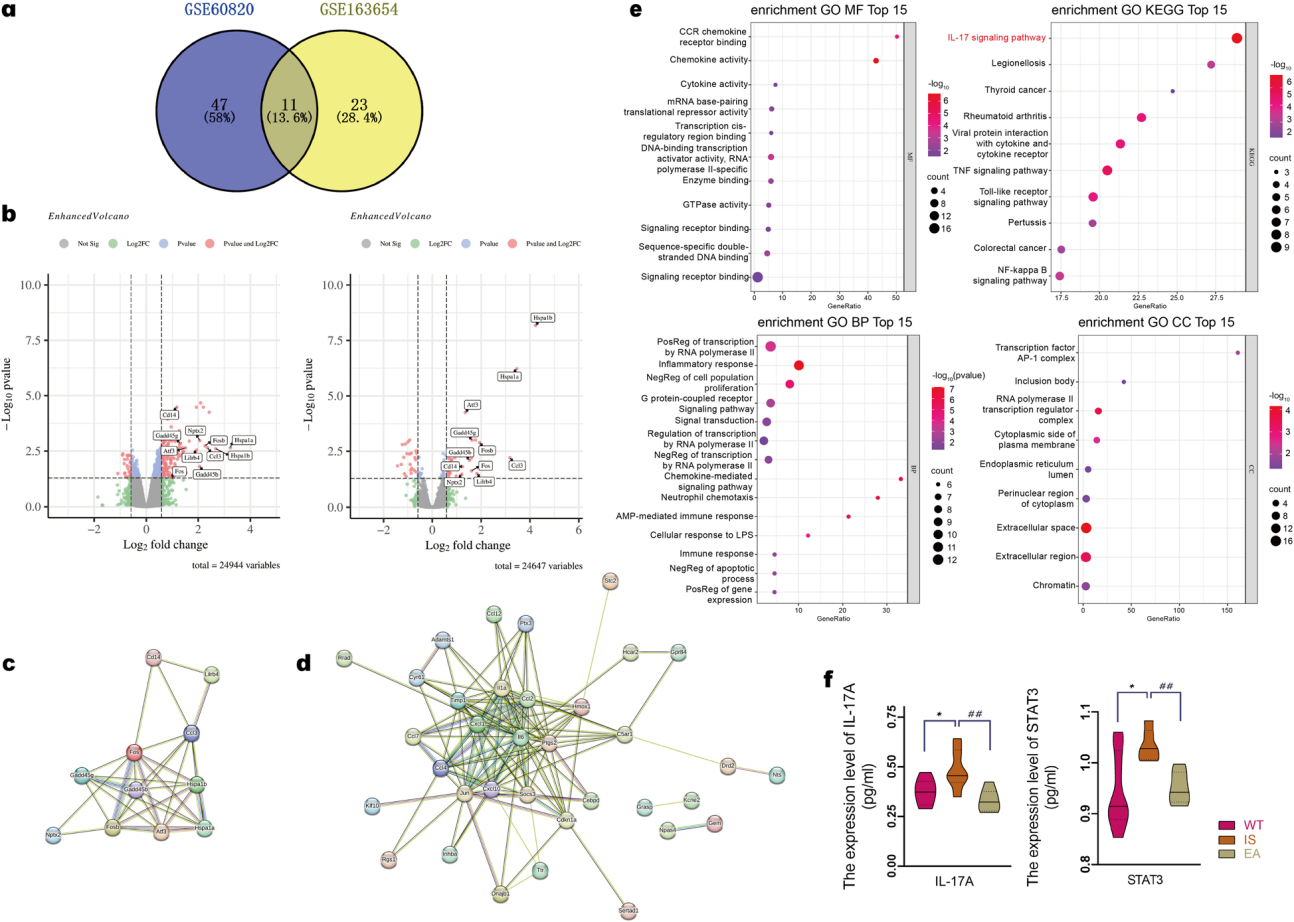
DEGs and pathway enrichment in IS models showing that differences in expression levels of IL-17A. **a** Venn diagram of datasets GSE60820 and GSE163654 showing 81 total DEGs and 11 common DEGs. **b** Volcano plots for GSE163654 (left) and GSE60820 (right). **c** Protein–protein interaction (PPI) network for intersecting DEGs. **d** PPI network for all DEGs. **e** GO and KEGG pathway enrichment analyses for DEGs. **f** ELISA analysis of the expression of IL-17A and STAT3. Left: IL-17A expression across groups after 14 days post-IS. Right: STAT3 expression levels (WT n = 8, IS n = 6, EA n = 8). Results are expressed as the mean ± SD, *p < 0.05 vs. WT; ## *p* < 0.01 vs. IS. Effect size: *t* test, 1.33 < Cohen’s d ≤ 2.68

Integrated analysis of the two datasets revealed a total of 81 DEGs, including 66 upregulated and 15 downregulated genes, with 11 overlapping DEGs identified via Venn diagram analysis (Fig. 2a). In GSE60820, 58 DEGs were detected (53 upregulated, 5 downregulated), among which *Cxcl1*, *Saa3*, *Ccl2*, *Fosb*, *Hspa1a*, *Ccl3*, *Hspa1b*, and *Gadd45b* exhibited significant differential expression (Fig. 2b, left). The GSE163654 dataset identified 34 DEGs (24 upregulated, 10 downregulated), with prominent changes observed in *Hspa1b*, *Hspa1a*, *Npas4*, and *Ccl3* (Fig. 2b, right).

Protein–protein interaction (PPI) networks were constructed using the STRING database to visualize interactions among all DEGs (Fig. 2d) and the intersecting DEGs (Fig. 2c). Functional enrichment analysis via the DAVID platform (Fig. 2e) revealed that DEGs were significantly enriched in multiple inflammatory and stress-related signaling pathways, including the IL-17A signaling pathway, TNF signaling pathway, MAPK signaling pathway, chemokine signaling pathway, and NF-κB signaling pathway.

It was observed that IL-17A and STAT3 expression levels were elevated in the IS group compared to the WT group (*p* < 0.05 for both; Fig. 2f). Notably, EA preconditioning significantly reduced the overexpression of the IL-17A induced by IS (*p* < 0.01 for both; Fig. 2f), suggesting that EA may exert neuroprotective effects by modulating IL-17A-related inflammatory signaling and promoting recovery post-stroke.

### EA alleviated the abnormal astrocytic calcium activity in the IS mice

**Fig. 3:**
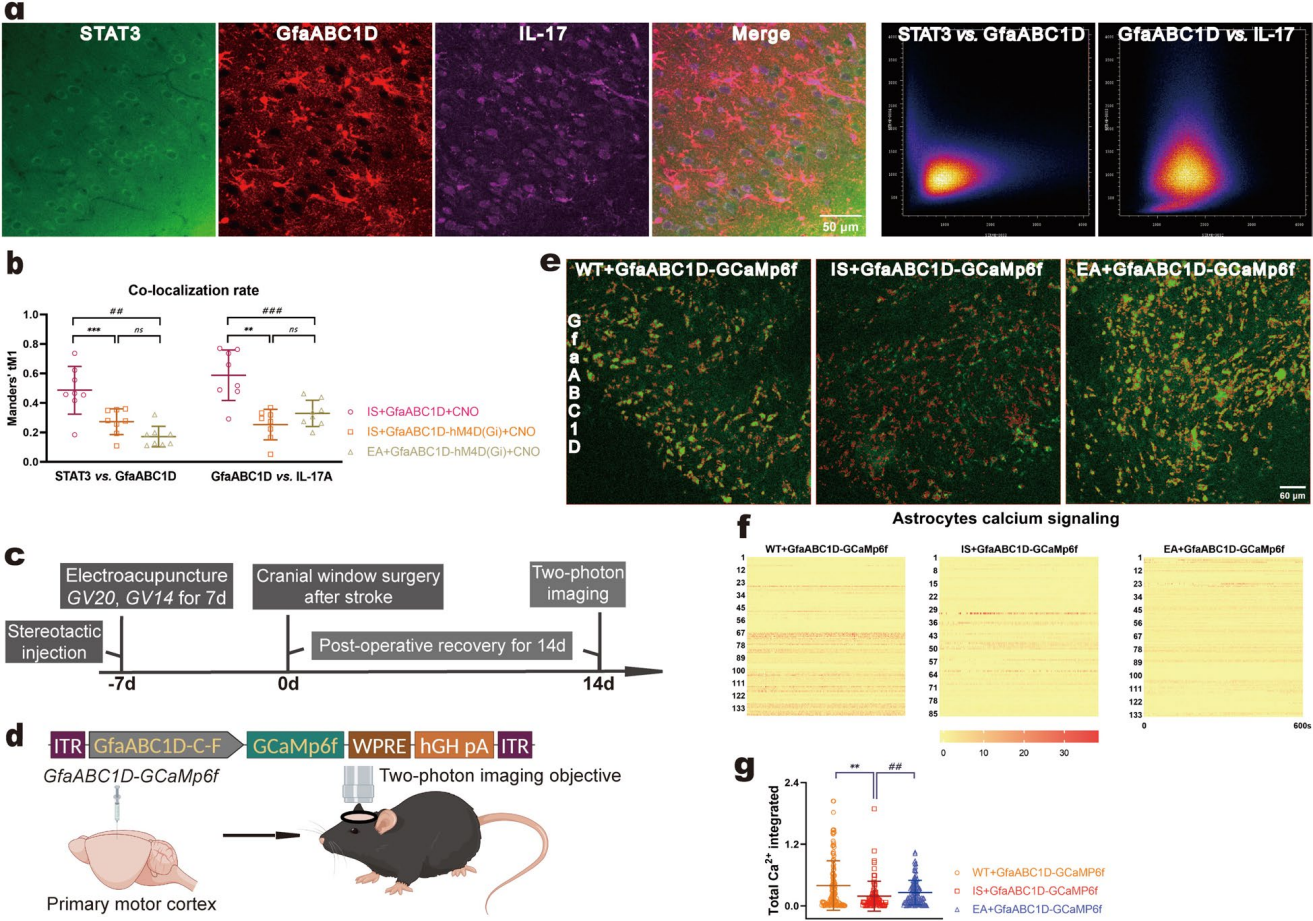
EA modulated both the IL-17A identified through IF in astrocytes and the altered calcium dynamics of astrocyte in IS mice observed via two-photon imaging. **a** Left: Immunofluorescence of the primary motor cortex after 14 days post-IS. Right: Pearson's *R* correlation analysis between STAT3/astrocytes and IL-17A/astrocytes in M1. **b** Co-localization rate of STAT3 vs. GfaABC1D and GfaABC1D vs. IL-17A in the M1 region of mice 14 days after IS (n = 8). ** *p* < 0.01 vs. IS + GfaABC1D + CNO, ***p < 0.001 vs. IS + GfaABC1D + CNO, ##*p* < 0.01 vs. EA + GfaABC1D-hM4D(Gi) + CNO, and ###*p* < 0.001 vs. EA + GfaABC1D-hM4D(Gi) + CNO. **c** Experimental timeline. **d** Top: Schematic of adeno-associated virus (rAAV-GfaABC1D-GCaMP6f-WPRE-pA). Bottom: Flow chart for two-photon calcium imaging. **e** Two-photon imaging showed 139 astrocyte ROIs in the M1 region in the WT + GfaABC1D-GCaMP6f group (4 mice), 86 astrocyte ROIs in the IS + GfaABC1D-GCaMP6f group (4 mice), and 134 astrocyte ROIs in the EA + GfaABC1D-GCaMP6f group (3 mice), the ROI of the area circled in red. **f** Thermograms depicting calcium activity in astrocytes from in the M1 region across three groups (n = 86–139). **g** Comparative filtered analysis of Total Ca^2^⁺ Integrated across groups (WT + GfaABC1D-GCaMP6f, IS + GfaABC1D-GCaMP6f, EA + GfaABC1D-GCaMP6f, n = 78–132). Results are expressed as the mean ± SD, **p < 0.01 vs. WT + GfaABC1D-GCaMP6f; ##*p* < 0.01 vs. IS + GfaABC1D-GCaMP6f. Effect size: Mann–Whitney test, 0.19 < r ≤ 0.87; *t* test, 1.64 < Cohen’s d ≤ 2.36

It was reported that the IL-17A could be secreted by the astrocyte [28]. In this case, the immunofluorescence analysis was used to confirmed that the GfaABC1D (a widely-used marker of astrocytes) was colocalized with STAT3 and IL-17A (Fig. 3a). To directly investigate the pathological changes of astrocyte during the stroke in vivo, we injected rAAV-GfaABC1D-GCaMP6f-WPRE-hGH polyA virus into the M1, and specifically detect the calcium activity on astrocyte through GCaMP6f. Compared to the WT group, the IS group exhibited a significant decline in Total Ca^2^⁺ Integrated. Following EA preconditioning, Total Ca^2^⁺ Integrated was significantly increased (Fig. 3b–f). The IS group showed a marked reduction in peak calcium level compared to the WT group, while the EA group showed a resumption post-intervention (Extended Fig. S3a). The results demonstrated that EA effectively counteracted the hypoactivity of astrocytes during stroke recovery.

### Inhibiting the astrocytic activity blocked EA-mediated behavioral improvement in IS mice

**Fig. 4:**
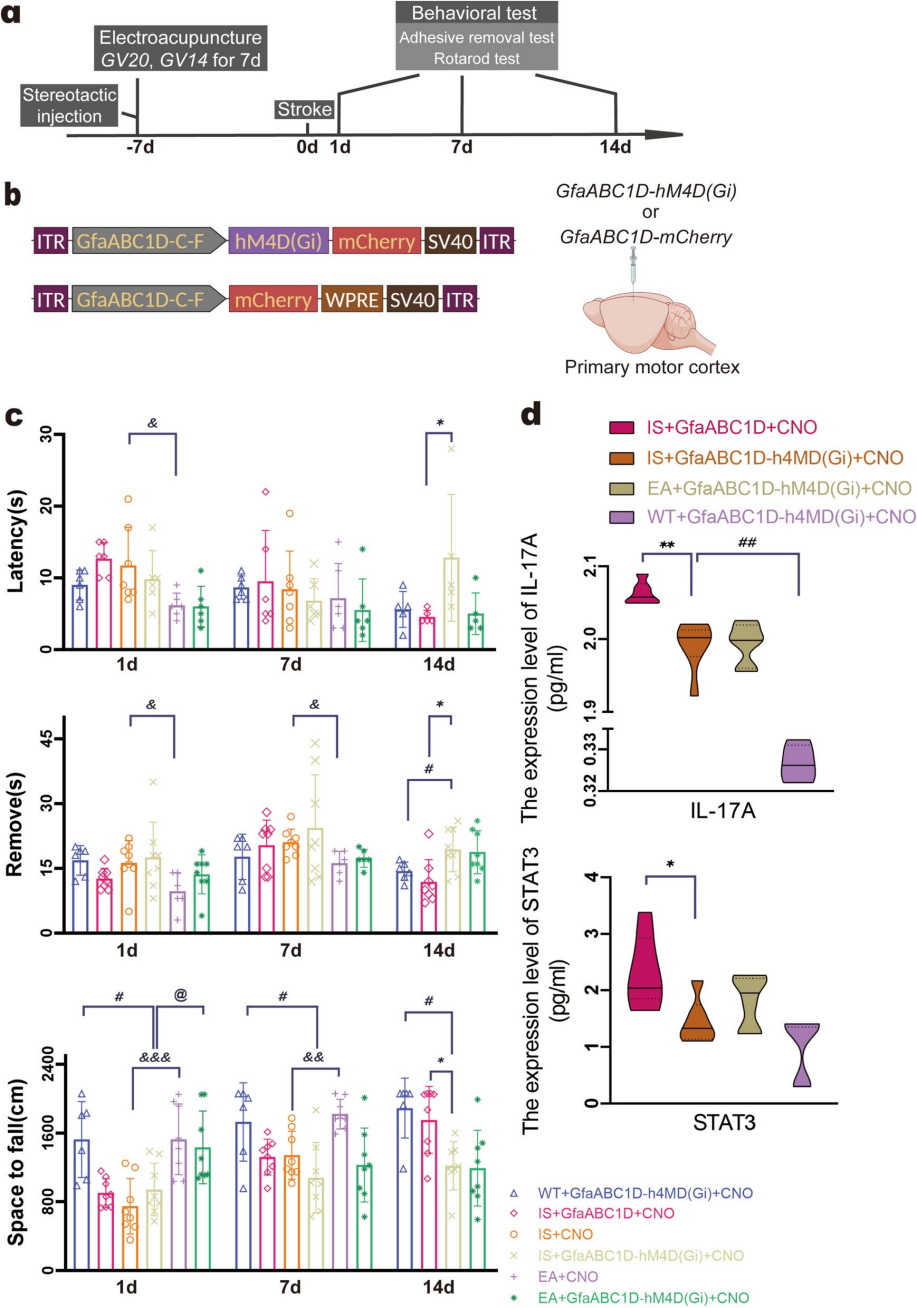
Astrocyte activity inhibition prevented EA-mediated improvement in IS mice, exhibiting congruent modulation of IL-17A expression trends. **a** Experimental timeline. **b** Schematic of adeno-associated viruses (rAAV-GfaABC1D-mCherry-WPRE-SV40 pA vs. rAAV-GfaABC1D-hM4D(Gi)-mCherry-SV40 pA) and stereotaxic virus injection into the primary motor cortex. **c** Detection latency and tear time of the Adhesive removal test, and total distance traveled of the Rotarod test (WT + GfaABC1D-hM4D(Gi) + CNO, IS + GfaABC1D + CNO, IS + CNO, IS + GfaABC1D-hM4D(Gi) + CNO, EA + CNO, EA + GfaABC1D-hM4D(Gi) + CNO, n ≥ 5). **d** IL-17A and STAT3 expression across groups post-IS (IS + GfaABC1D + CNO, IS + GfaABC1D-hM4D(Gi) + CNO, EA + GfaABC1D-hM4D(Gi) + CNO, WT + GfaABC1D-hM4D(Gi) + CNO, n ≥ 6). Results are expressed as the mean ± SD, * *p* < 0.05, ** *p* < 0.01 vs. IS + GfaABC1D; # *p* < 0.05, ## *p* < 0.01 vs. WT + GfaABC1D-hM4D(Gi); & *p* < 0.05, && *p* < 0.01, &&& *p* < 0.001 vs. IS; @ *p* < 0.05 vs. IS + GfaABC1D-hM4D(Gi). Effect size: Mann–Whitney test, 0.41 < r ≤ 0.96; Welch *t* test, 0.23 < Cohen’s d ≤ 1.57; *t* test, 0.12 < Cohen’s d ≤ 2.11

To further confirm the role of astrocyte in the stroke or EA preconditioning, chemogenetically inhibition of astrocytic activity by injecting rAAV-pZac2.1 GfaABC1D-hM4D (Gi)-mCherry-SV40 poly into the M1, and CNO was injected intraperitoneally to activate the Gi channels. Which is known to suppress the cAMP-PKA pathway and downstream calcium channel/pump phosphorylation [29], ultimately driving the observed modulation of astrocytic activity (Fig. 4a, b). Compared to IS + GfaABC1D group, the Total Ca^2^⁺ Integrated was significantly declined in IS + GfaABC1D-hM4D (Gi) group, also exhibited a significant turndown in Peak Value, consistent with the trend observed in Total Ca^2^⁺ Integrated (Extended Fig. S2, Extended Fig. S3b). These results validated the presence of calcium inactive in astrocyte within the IS + GfaABC1D-hM4D (Gi) group.

In the behavioral experiments, it showed that there was no significant difference in the IS + GfaABC1D compared to the IS + GfaABC1D-hM4D (Gi) group (Fig. 4c), confirming viral construct neutrality in baseline pathophysiology. At 14 days post-ischemia, IS + GfaABC1D-hM4D (Gi) group mice exhibited prolonged adhesive detection latency (Fig. 4c, top), delayed paper removal (Fig. 4c, middle), and reduced rotarod performance (Fig. 4c, bottom) versus control. These findings demonstrated that inhibition of astrocytic activity might further aggravate the stroke-related pathologies. Furthermore, EA failed to ameliorate these deficits in hM4D (Gi)-activated mice (Fig. 4c), confirming intact astrocytic activity as an essential condition for EA-mediated therapeutic efficacy in stroke recovery. Behavioral phenotypes, astrocytic calcium suppression and astrocytic functional impairment were observed in the context of both hM4D(Gi) expression and CNO administration exclusively.

Next, the expression of IL-17A and STAT3 was examined after inhibition of astrocytic activity. The results showed that the expression of IL-17A and STAT3 was significantly reduced in the IS mice with chemoinhibition of astrocytic activity. Compared to control vectors, as quantified by ELISA optical density measurements (Fig. 4d). Moreover, the EA’s modulation of IL-17A or STAT3 was blocked by inhibiting of astrocytic activity (Fig. 4d). Altogether, these results suggested that the importance role of astrocytic activity in the pathogenesis of stroke and EA-mediated effect.

### Overexpression of IL-17A in astrocyte mimicked the dysregulated calcium activity in the IS

**Fig. 5:**
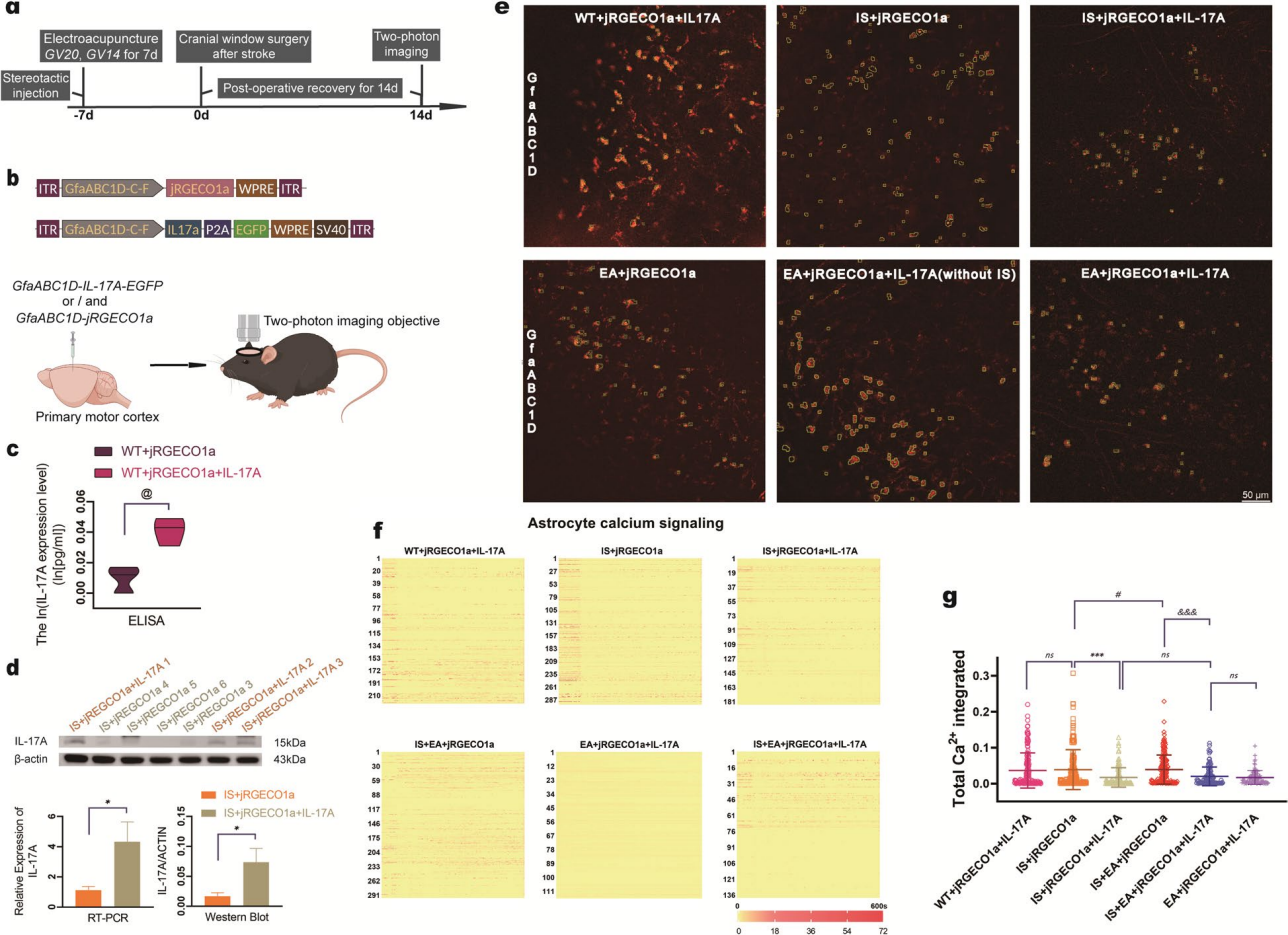
Astrocyte-specific IL-17A overexpression in WT mice recapitulated the aberrant calcium signaling dynamics characteristic of astrocytes in IS models. **a** Experimental timeline. **b** Top: Schematic of adeno-associated viruses (rAAV-GFaABC1D-IL-17A-EGFP-SV40 pA vs. rAAV-GfaABC1D-jRGECO1a-WPRE-pA). Bottom: Flow chart for two-photon calcium imaging. **c** Overexpression of IL-17A on astrocyte was verified by ELISA in WT mice. **d** Overexpression of IL-17A on astrocyte was verified by RT-PCR and Western Blot in IS + jRGECO1a + IL-17A mice. **e** Two-photon imaging showed 222 astrocyte ROIs in the M1 region in the WT + jRGECO1a + IL-17A group (3 mice), 296 astrocyte ROIs in the IS + jRGECO1a group (3 mice), 184 astrocyte ROIs in the IS + jRGECO1a + IL-17A group (3 mice), 296 astrocyte ROIs in the IS + EA + jRGECO1a group (3 mice), 116 astrocyte ROIs in the EA + jRGECO1a + IL-17A group (3 mice), and 138 astrocyte ROIs in the IS + EA + jRGECO1a + IL-17A group (3 mice), the ROI of the area circled in yellow. **f** Thermograms depicting calcium activity across five groups (n = 116–296). **g** Comparative filtered analysis of Total Ca^2^⁺ Integrated in WT + jRGECO1a + IL-17A, IS + jRGECO1a, IS + jRGECO1a + IL-17A, IS + EA + jRGECO1a, EA + jRGECO1a + IL-17A and IS + EA + jRGECO1a + IL-17A groups (n = 89–189). Results are expressed as the mean ± SD, @ *p* < 0.05 vs. WT + jRGECO1a; *p < 0.05, ***p < 0.001 vs. IS + jRGECO1a; #*p* < 0.05 vs. IS + EA + jRGECO1a; &&& *p* < 0.001 vs. IS + EA + jRGECO1a + IL-17A. Effect size: Mann–Whitney test, 0.12 < r ≤ 0.45; Welch *t* test, 0.23 < Cohen’s d ≤ 3.22; *t* test, 0.81 < Cohen’s d ≤ 3.49

Above results indicated the IL-17A in astrocyte might play a critical role during in the stroke or EA-mediated effect. Thus, to further confirm the function of IL-17A on astrocyte, we overexpressed IL-17A in the astrocytes of WT mice by injecting the rAAV-GfaABC1D-IL17A-P2A-EGFP-WPRE-SV40 polyA virus into the M1 region of WT mice, as validated by ELISA (Fig. 5a–c). In addition, RT-PCR and Western Blot experiments also showed the enhanced expression of IL-17A in the IS + jRGECO1a + IL-17A mice (Fig. 5d). Most importantly, viral-mediated IL-17A expression recapitulated pathological calcium dynamics observed in IS mice, as comparable Total Ca^2^⁺ Integrated were shown between IL-17A-overexpressing WT + jRGECO1a + IL-17A and IS + jRGECO1a groups (Fig. 5e–g). In the IS + jRGECO1a + IL-17A group, the calcium activity of astrocytes was lower than that in the IS + jRGECO1a group, and the IS + EA + jRGECO1a + IL-17A group showed that the effect of EA was also blocked after overexpression of IL-17A.

**Fig. 6:**
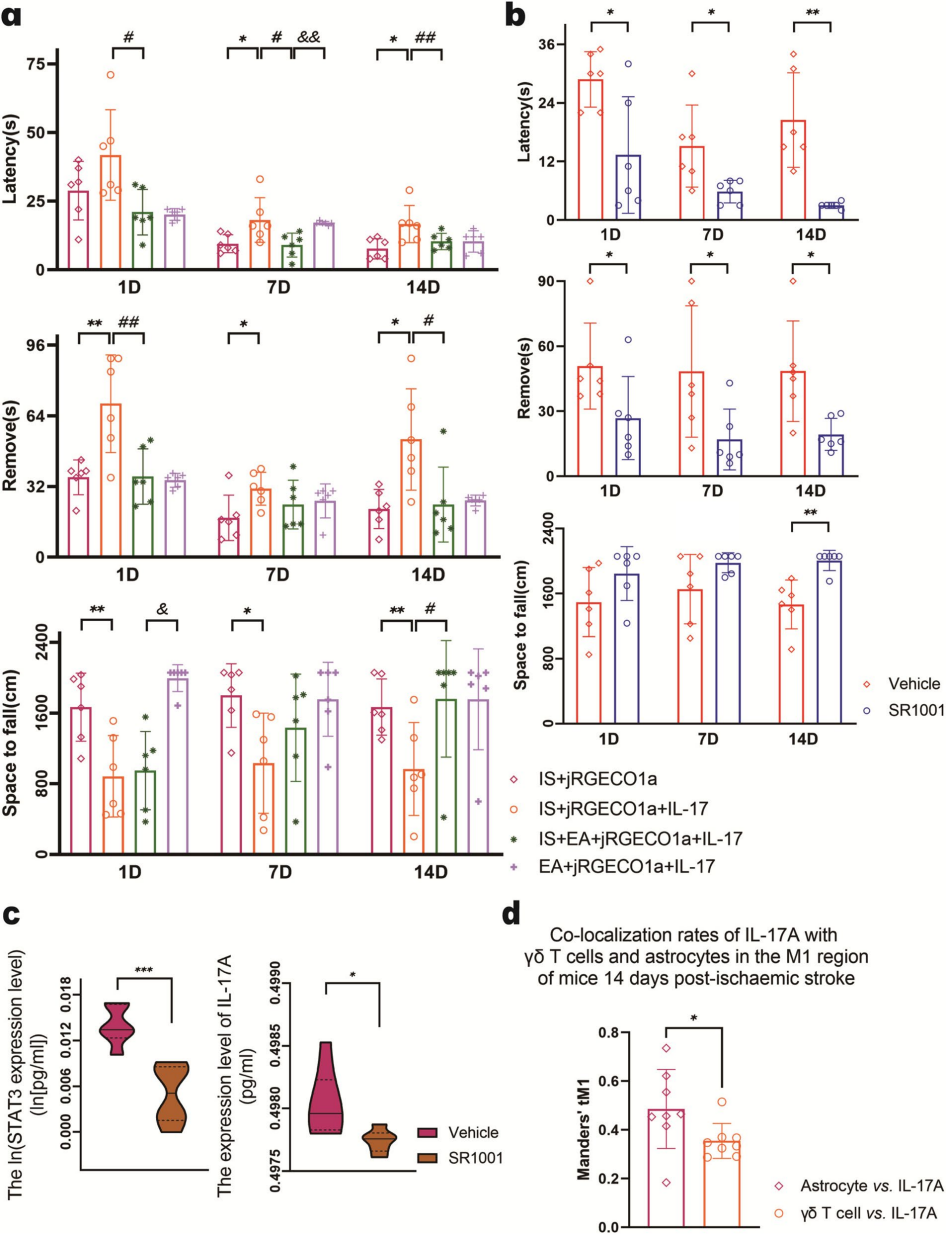
Behavioral tests for inhibiting IL-17A and overexpressing IL-17A. **a** Detection latency and tear time of the Adhesive removal test, and total distance traveled of the Rotarod test (IS + jRGECO1a, IS + jRGECO1a + IL-17A, and EA + jRGECO1a + IL-17A; n = 6; *p < 0.05, **p < 0.01 vs. IS + jRGECO1a; #*p* < 0.05, ##*p* < 0.01 vs. IS + jRGECO1a + IL-17A). **b** Detection latency and tear time of the Adhesive removal test, and total distance traveled of the Rotarod test (Vehicle, SR1001; n = 6; *p < 0.05, **p < 0.01 vs. Vehicle). **c** IL-17A and STAT3 expression across groups post-IS 14 days (Vehicle, SR1001; n = 6; *p < 0.05, **p < 0.01, ***p < 0.001 vs. Vehicle). **d** Co-localization rates of IL-17A with γδ T cell and astrocyte in the M1 region of mice 14 days post-IS(n = 8). Results are expressed as the mean ± SD. Effect size: Mann–Whitney test, 0.62 < r ≤ 0.94; *t* test, 1.39 < Cohen’s d ≤ 2.54

Meanwhile, overexpression of IL-17A in astrocyte aggravated the sensorimotor dysfunction caused by IS, the IS + jRGECO1a + IL-17A group significantly prolonged removal times in the adhesive removal test while reducing total distance traveled in the rotarod test at 1, 7, and 14 days post-IS compared to the IS + jRGECO1a control (Fig. 6a), the latency time of the adhesive paper was also prolonged on the 7th and 14th days after IS, which could be shorten by EA preconditioning. Noteworthy, in addition to the astrocyte, IL-17A could be produced by γδ T cells, Th17 cells, and infiltrating myeloid cells [30]. In this case, SR1001, a widely used inhibitor of RORγt, an upstream transcription factor of IL-17A in the IS mice, was employed. It was observed from the ELISA analysis that the expression of IL-17A was significantly decreased by the SR1001 (Fig. 6c). Furthermore, the IS mice with SR1001 mice showed a great improvement demonstrated by the behavioral tests (Fig. 6b). Although these results suggested the importance of IL-17A during the stroke, there was a higher co-localization of IL-17A and astrocyte compared to that of γδ T cells. These results indicated astrocyte might the main source of IL-17A in the condition set by this study.

Together, these results suggested that the expression/function of IL-17A play a crucial role in the astrocytic activity, thus might contribute to the pathogenesis of stroke or EA-mediated improvement.

## Discussion

This study revealed that EA at GV20 and GV14 could improve the sensorimotor function after stroke by modulating the astrocytic calcium activity in which was associated with the IL-17A. The regulatory effect of EA was prevented by the inhibiting the astrocytic activity. Besides that, overexpression of IL-17A in the physiological mice could mimic the abnormal calcium signaling dynamics represented in stroke mice. Building on prior studies regarding EA’s acute suppression of IL-17A and neurotoxic astrocytes [22], while this study focused on the recovery phase of stroke. We found that inhibition of astrocytic activity decreased IL-17A but hindered functional recovery, suggesting that preserving astrocytic integrity is crucial. Through in vivo calcium imaging and bidirectional manipulation, we aimed to complement previous endpoint-level reports by providing additional insights into these recovery dynamics.

In this study, it was showed an attenuated astrocytic calcium activity after overexpression of IL-17A, while a decrease of IL-17A expression was observed after chemoinhibition of astrocyte. These results indicated that there might be a feedback loop between the IL-17A and astrocytic activity. The previous studies showed that IL-17A could influence the astrocytic activity via IL-17RA/STAT3/SOCS3 and Src/PI3K/NF-κB pathway [31, 32]. We observed that there was no significant difference in the expression level of IL-17A between the mice treated with EA plus inhibition of astrocytic activity and that with inhibition of astrocytic activity alone (Fig. 4d), suggesting that the intact function of astrocytic activity was required in the EA-mediated effect of the regulation of IL-17A. However, the exact role of feedback loop between IL-17A and astrocytes during the stroke or EA-mediated effect should be explored in depth in the future.

It was noteworthy that IL-17A could be also released by microglia, in which IL-17A acts as a regulator modulating the activation states, inflammatory cytokine production, and neuroimmune interactions, impacting cerebral pathology and immune homeostasis [33, 34]. To exclude the potential involvement of IL-17A from microglia, we performed immunofluorescence experiments to detect the expression of IL-17A on the microglia. The results showed that microglia were not significantly active in the M1 of IS mice 7 days post-stroke (Extended Data: S1c). The reason for the inactivity of microglia here may be that microglia usually activate rapidly within minutes to hours after IS (type M1) and peak at 3–5 days before gradually subsiding or transforming to a repair phenotype (type M2) [35]. While during the Day 7 after stroke in this study, microglia activation might have entered the regression phase. However, the activation of astrocyte usually increased immediately after ischemic injury and gradually decreases within several weeks [36]. It was speculated that the peak of astrocytic activation may be ranged from 4 to 14 days [37]. The co-localized of GfaABC1D and IL-17A was consistent with this timeline (Fig. 3a). Thus, it is possible that the role of microglia in the stroke pathologies or EA-mediated effect is not as prominent as that of astrocyte in this study. However, it could be hypothesized that IL-17A released from microglia was also involved in the pathological process after IS during the acute period of IS, which it requires us to conduct subsequent research.

It was reported that IL-17A levels were elevated after day 3, which was derived mostly from γδ T cell infiltration peaks within 72 h post-stroke [38], while astrocyte activation escalates over days 4–14 [37]. Moreover, we used SR1001, which has a pharmacological mechanism strongly correlated with γδ T cells, to continuously inhibit IL-17A in mice 7 days before IS and observed the recovery of sensorimotor ability in mice 14 days after IS. According to the results in Fig. 6b, c, inhibited IL-17A alone with SR1001 before IS can effectively restore sensorimotor ability of mice during the recovery period of IS. Combined with the results in Fig. 6d, during the recovery period after IS, the co-localization rate of γδ T cells and IL-17A was lower than that of astrocyte, proving that astrocyte dominated the activity of IL-17A 14 days after IS. Thus, while multiple cell types secrete IL-17A in stroke, their contributions might be phase-dependent and interactive. Due to time constraints, this theory lacks more experimental support, and these aspects will also be the focus of our subsequent research.

In this study, EA preconditioning for the stroke therapy was implemented, and this preventive intervention was different from the most pattern in the clinical practice or other pre-clinical experiments, in which the intervention was administrated after the stroke occurrence [39]. However, there are mainly two reasons for us to adopt this early prevention for stroke. First, there is an obvious limitation of the techniques used in the study. Cranial window implantation for in vivo two-photon imaging requires surgical removal of mice scalp containing the GV20 and GV14 acupoints. Thus, the EA at GV20 and GV14 was administrated before the cranial window implantation/surgery. Second, EA preconditioning was used for improving immunity and reducing the risk of disease occurrence, previous studies indicate that EA preconditioning applied with systemic inflammation by lipopolysaccharide could show its anti-inflammatory effects [40], and there were other evidence shown that EA preconditioning at GV20 and GV14 improved motor function recovery and mitigated pathological damage to brain tissue in IS [41, 42], which highlighted the neuroprotective potential of EA preconditioning.

Additionally, our findings position EA at GV20 and GV14 as a unique non-invasive strategy that acts through an astrocyte and IL-17A. This mechanism contrasts with and potentially complements other prominent neuromodulation approaches. For instance, transcranial direct current stimulation (tDCS) primarily exerts its effects by modulating neuronal membrane potentials and synaptic plasticity, with its anti-inflammatory effects being a secondary consequence [43]. Conventional rehabilitation training enhances functional recovery through activity-dependent cortical remapping and strengthening of existing neural circuits [44]. In contrast, our data suggest that EA could directly targets the neuroimmune, specifically modulating astrocytic calcium activity and the downstream IL-17A to create a pro-repair microenvironment. This direct impact on glial cells may explain why EA can produce benefits even as a preconditioning intervention.

In summary, this study revealed the role of IL-17A on astrocyte was important in the EA-mediated improvement of sensorimotor function after stroke. This provides a new theoretical basis for the molecular mechanism of traditional Chinese medicine in the treatment of ischemic stroke and also points out the direction for the development of new intervention strategies targeting astrocyte-related inflammatory signals in the future.

## Research limitations and future perspectives

Several important limitations warrant acknowledgment. First, the precision of the IL-17A cellular source remains incomplete. While immunofluorescence demonstrates astrocytic IL-17A co-localization, single-cell RNA sequencing and astrocyte-specific IL-17A knockout approaches would help clarify the contributions of IL-17A across diverse cellular sources. This represents an essential next step for comprehensive cellular mapping.

Second, downstream mechanistic pathways require elucidation. IL-17A likely engages STAT3, NF-κB, and MAPK signaling cascades to regulate astrocytic excitability [45, 46]. These pathways plausibly modulate L-type and P/Q-type calcium channel phosphorylation via PKA-dependent mechanisms, thereby controlling calcium homeostasis [47–50]. Pharmacological inhibition of these signaling nodes and astrocyte-selective gene knockdown approaches are critical for mechanistic validation.

Third, our investigation focuses on the subacute recovery window (days 14 post-stroke). IL-17A and astrocytic calcium dynamics exhibit marked temporal heterogeneity. Longitudinal two-photon imaging and staged molecular profiling across acute, subacute, and chronic phases would comprehensively delineate this pathway's evolving role.

Finally, EA likely engages both IL-17A-dependent and IL-17A-independent mechanisms. ATP release, alternative cytokines (TNF-α, IL-6), and astrocyte–neuron metabolic coupling (lactate shuttle) represent promising alternative mediators [51–54]. Validating these candidate mechanisms, however, requires rigorous dissection via pharmacological inhibitors, genetic tools, and refined functional assays constitutes an essential requirement for a comprehensive understanding of electroacupuncture's multifaceted biology. These future investigations will substantially advance our mechanistic understanding of neuroimmune modulation in stroke recovery.

## Supplementary Information

Additional file1 (PZFX 832 KB)

Additional file2 (PZFX 307 KB)

Additional file3 (ZIP 164032 KB)

Additional file4 (TIF 2316 KB)

**Additional file5: Extended Data Fig. 1.** **a** Immunofluorescence in the primary motor cortex at 14 days post-IS [IS+GfaABC1D-hM4D(Gi), EA+GfaABC1D-hM4D(Gi)]. **b** Pearson's R correlation analysis between STAT3/astrocyte and IL-17A/astrocyte in IS+GfaABC1D-hM4D(Gi), EA+GfaABC1D-hM4D(Gi) groups using Fiji. **c** Immunofluorescence after 7 days post-IS showing that microglia quantity was not evident. **d** Pearson’s R correlation data for IS, IS+GfaABC1D-hM4D(Gi), EA+GfaABC1D-hM4D(Gi) group (n = 3). **e** Immunofluorescence after 14 days post-IS showing that γδ T cell quantity was not evident.

**Additional file6: Extended Data Fig. 2.** **a** Flow chart for two-photon calcium imaging. **b** Two-photon imaging showed 287 astrocyte ROIs in the M1 region in the IS+GCaMP6f+CNO group (4 mice), 175 astrocyte ROIs in the IS+GCaMP6f+hM4D(Gi)+CNO group (4 mice), 53 astrocyte ROIs in the EA+GCaMP6f+hM4D(Gi)+CNO group (3 mice), and 219 astrocyte ROIs in the WT+GCaMP6f+hM4D(Gi)+CNO group (3 mice). **c** Thermograms depicting calcium activity across groups (n = 53–287). **d** Comparative filtered analysis of Total Ca²⁺ Integrated in IS+GCaMP6f+CNO, IS+GCaMP6f+hM4D(Gi)+CNO, EA+GCaMP6f+hM4D(Gi)+CNO groups, and WT+GCaMP6f+hM4D(Gi)+CNO (n = 47–264). Results are expressed as the mean ± SD, $$$ *p *< 0.001 vs. IS+GCaMP6f+CNO; × *p *< 0.05 vs. IS+GCaMP6f+hM4D(Gi)+CNO.

**Additional file7: Extended Data Fig. 3.** **a** Comparative filtered analysis of Peak Value, and Frequency across WT+GfaABC1D-GCaMP6f, IS+GfaABC1D-GCaMP6f, and EA+GfaABC1D-GCaMP6f groups (n = 78–132). **b** Comparative filtered analysis of Peak Value, Frequency in IS + GCaMP6f + CNO, IS+GCaMP6f + hM4D(Gi) + CNO, EA+GCaMP6f+hM4D(Gi)+CNO groups, and WT+GCaMP6f+hM4D(Gi)+CNO (n = 47–264). **c** Comparative filtered analysis of Peak Value, Frequency in WT+jRGECO1a+IL-17A, IS+jRGECO1a, IS+jRGECO1a+IL-17A, IS+EA+jRGECO1a, IS+EA+jRGECO1a+IL-17A and EA+jRGECO1a+IL-17A groups (n = 89–189). Results are expressed as the mean ± SD, *p < 0.05, **p < 0.01, *** *p *< 0.001 vs. WT+GfaABC1D-GCaMP6f; ## *p* < 0.01 vs. IS+GfaABC1D-GCaMP6f; $$$ *p* < 0.001 vs. IS+GCaMP6f+CNO; ××× *p *< 0.001 vs. IS+GCaMP6f+hM4D(Gi)+CNO; &&& *p* < 0.001 vs. IS+jRGECO1a+IL-17A; @@@ *p* < 0.001 vs. IS+jRGECO1a; ∆∆ *p *< 0.01 vs. IS+EA+jRGECO1a; %%% *p* < 0.001 vs. EA+jRGECO1a+IL-17A.

## Data Availability

The data that support the findings of this study are available from the corresponding authors upon reasonable request.

## Abbreviations

AAV: Adeno-associated virus
AMPK: AMP-activated protein kinase
ANOVA: Analysis of variance
AP: Anterior–posterior (stereotaxic coordinate)
ATP: Adenosine triphosphate
BDNF: Brain-derived neurotrophic factor
BP: Biological process (Gene Ontology category)
cAMP: Cyclic adenosine monophosphate
CC: Cellular component (Gene Ontology category)
CNO: Clozapine-N-oxide
CXCL1: C-X-C motif chemokine ligand 1
DAPI: 4′,6-diamidino-2-phenylindole
DAVID: Database for Annotation, Visualization and Integrated Discovery
DEGs: Differentially expressed genes
DMSO: Dimethyl sulfoxide
EA: Electroacupuncture
EGFP: Enhanced green fluorescent protein
ELISA: Enzyme-linked immunosorbent assay
GBD: Global Burden of Disease
GCaMP6f: Genetically encoded calcium indicator (fast variant)
GEO: Gene Expression Omnibus
GfaABC1D: Compact glial fibrillary acidic protein (GFAP) promoter (astrocyte-specific)
GM-CSF: Granulocyte-macrophage colony-stimulating factor
GO: Gene Ontology
GV: Governing Vessel (acupoint meridian; GV14 Dazhui, GV20 Baihui)
hGH: Human growth hormone
hM4D(Gi): Gi-coupled human M4 muscarinic Designer Receptors Exclusively Activated by Designer Drugs
IL-6: Interleukin-6
IL-17A: Interleukin-17A
IL-23: Interleukin-23
IS: Ischemic stroke
jRGECO1a: Red genetically encoded calcium indicator
KEGG: Kyoto Encyclopedia of Genes and Genomes
M1: Primary motor cortex
M1FL: Forelimb region of the primary motor cortex
MAPK: Mitogen-activated protein kinase
MCAO/R: Middle cerebral artery occlusion/reperfusion
MF: Molecular function (Gene Ontology category)
ML: Mediolateral (stereotaxic coordinate)
NF-κB: Nuclear factor-κB
OCT: Optimal cutting temperature (compound)
OD: Optical density
P2A: Self-cleaving 2A peptide
PBS: Phosphate-buffered saline
PFA: Paraformaldehyde
PKA: Protein kinase A
pMCAO: Permanent middle cerebral artery occlusion
PPI: Protein–protein interaction
rAAV: Recombinant adeno-associated virus
ROIs: Regions of interest
RORγt: Retinoic acid receptor-related orphan receptor gamma t
RT-PCR: Reverse transcription–polymerase chain reaction
SREBPs: Sterol regulatory element-binding proteins
STAT3: Signal transducer and activator of transcription 3
STRING: Search Tool for the Retrieval of Interacting Genes/Proteins
SV40: Simian virus 40
tDCS: Transcranial direct current stimulation
TNF-α: Tumor necrosis factor-α
WPRE: Woodchuck hepatitis virus posttranscriptional regulatory element
WT: Wild type
γδ T cells: Gamma-delta T cells
