# Supplementary figures and images for: The IL-17A modulating astrocytic activity was associated with the electroacupuncture-mediated improvement of sensorimotor ability after stroke
Source: Chin Med. 2026 Jun 29;21:175. doi: 10.1186/s13020-026-01446-5 (PMC13312517; doi:10.1186/s13020-026-01446-5)

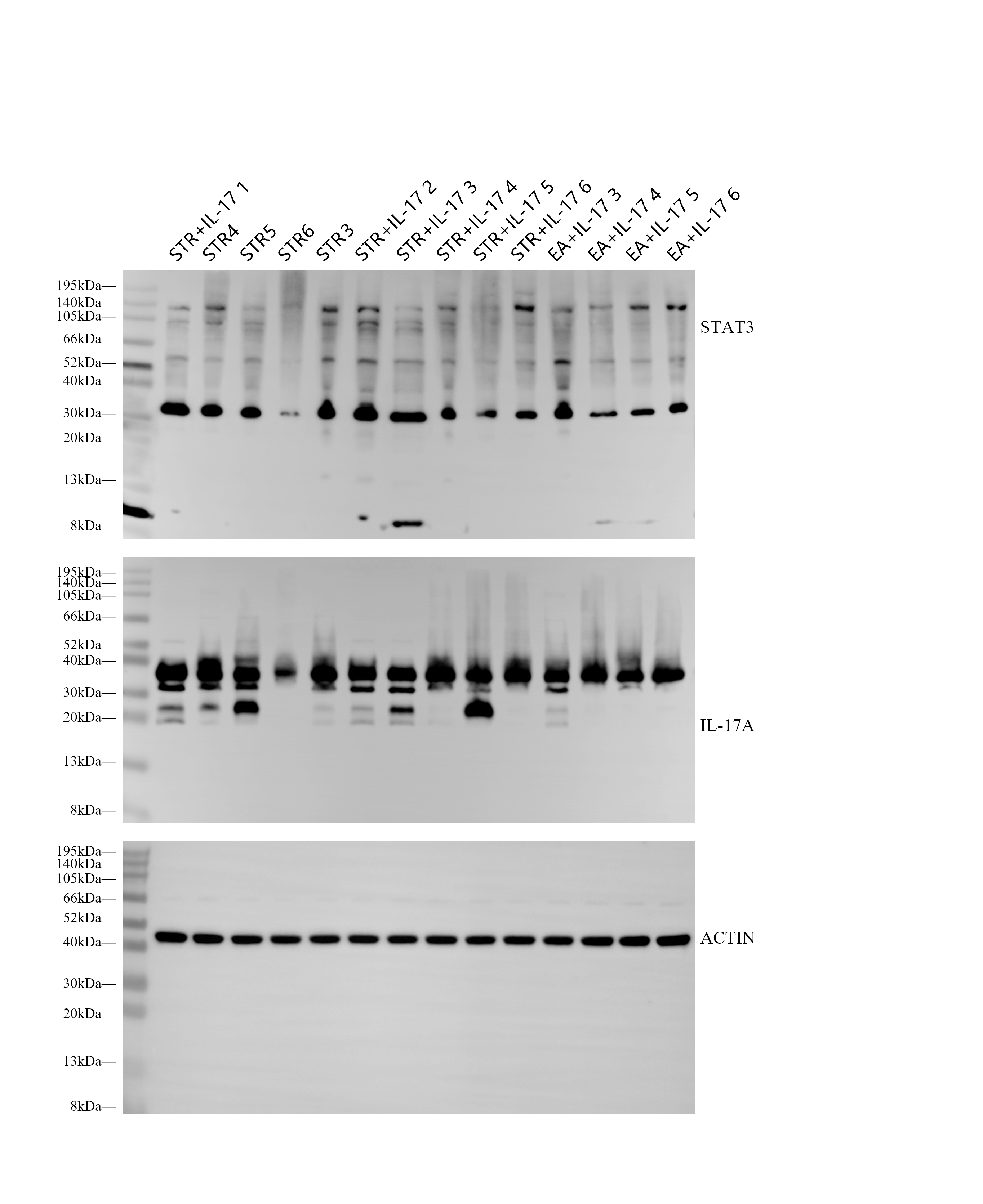

Supplement: Supplementary file 4 — Additional file4 (TIF 2316 KB) [file 13020_2026_1446_MOESM4_ESM.tif]

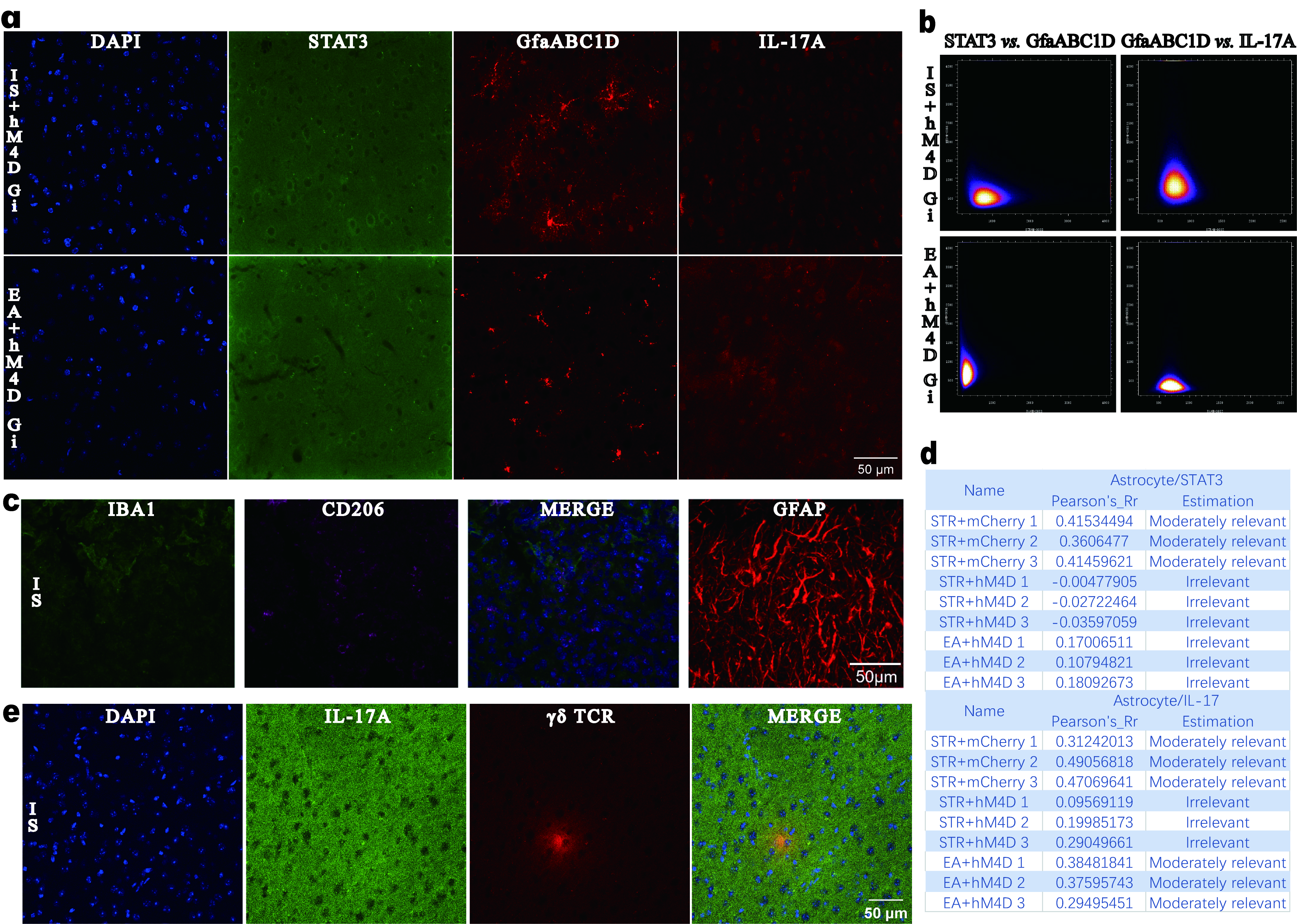

Supplement: Supplementary file 5 — Additional file5: Extended Data Fig. 1. a Immunofluorescence in the primary motor cortex at 14 days post-IS [IS+GfaABC1D-hM4D(Gi), EA+GfaABC1D-hM4D(Gi)]. b Pearson's R correlation analysis between STAT3/astrocyte and IL-17A/astrocyte in IS+GfaABC1D-hM4D(Gi), EA+GfaABC1D-hM4D(Gi) groups using Fiji. c Immunofluorescence after 7 days post-IS showing that microglia quantity was not evident. d Pearson’s R correlation data for IS, IS+GfaABC1D-hM4D(Gi), EA+GfaABC1D-hM4D(Gi) group (n = 3). e Immunofluorescence after 14 days post-IS showing that γδ T cell quantity was not evident. [file 13020_2026_1446_MOESM5_ESM.tif]

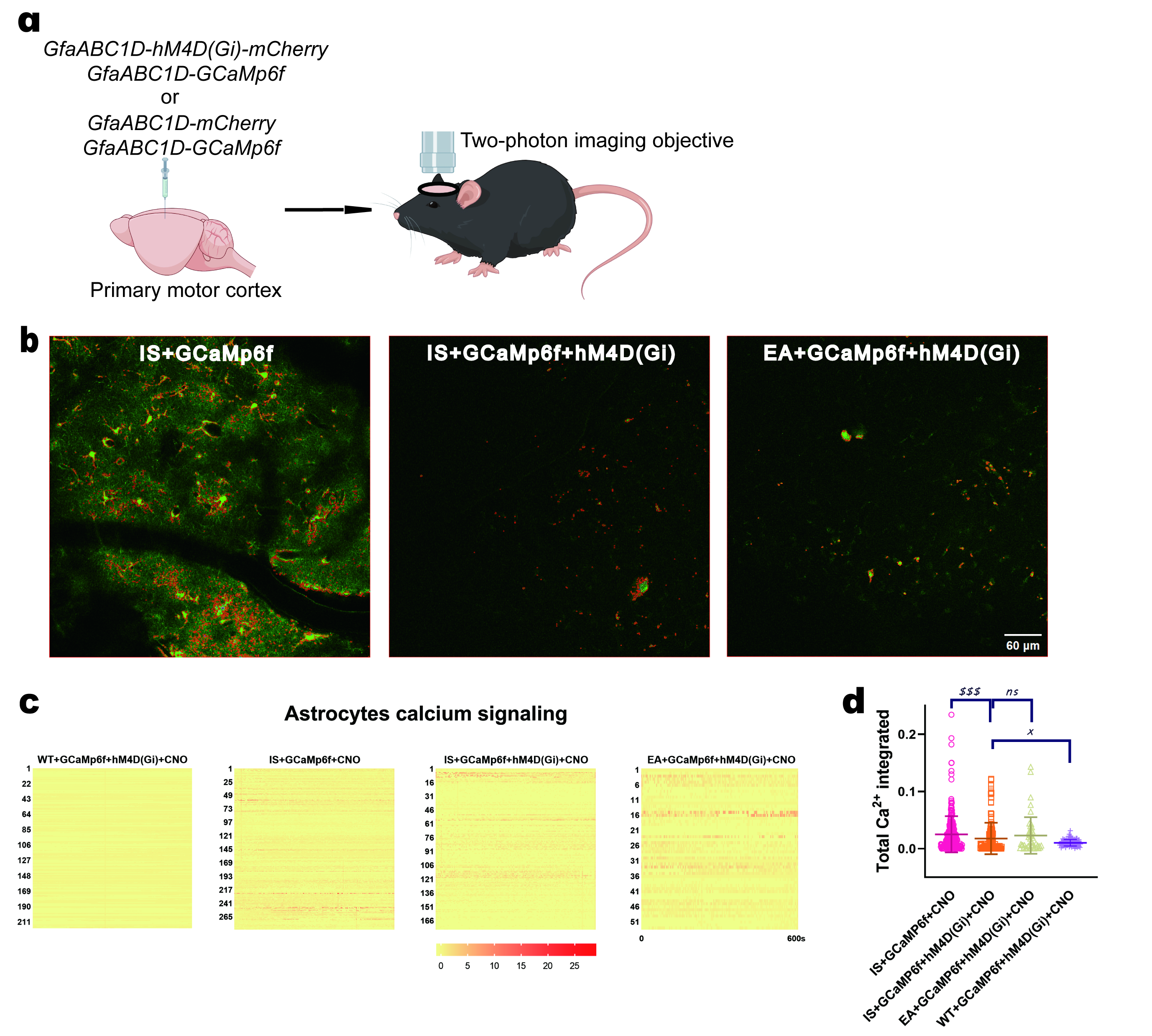

Supplement: Supplementary file 6 — Additional file6: Extended Data Fig. 2. a Flow chart for two-photon calcium imaging. b Two-photon imaging showed 287 astrocyte ROIs in the M1 region in the IS+GCaMP6f+CNO group (4 mice), 175 astrocyte ROIs in the IS+GCaMP6f+hM4D(Gi)+CNO group (4 mice), 53 astrocyte ROIs in the EA+GCaMP6f+hM4D(Gi)+CNO group (3 mice), and 219 astrocyte ROIs in the WT+GCaMP6f+hM4D(Gi)+CNO group (3 mice). c Thermograms depicting calcium activity across groups (n = 53–287). d Comparative filtered analysis of Total Ca²⁺ Integrated in IS+GCaMP6f+CNO, IS+GCaMP6f+hM4D(Gi)+CNO, EA+GCaMP6f+hM4D(Gi)+CNO groups, and WT+GCaMP6f+hM4D(Gi)+CNO (n = 47–264). Results are expressed as the mean ± SD, $$$ p < 0.001 vs. IS+GCaMP6f+CNO; × p < 0.05 vs. IS+GCaMP6f+hM4D(Gi)+CNO. [file 13020_2026_1446_MOESM6_ESM.tif]

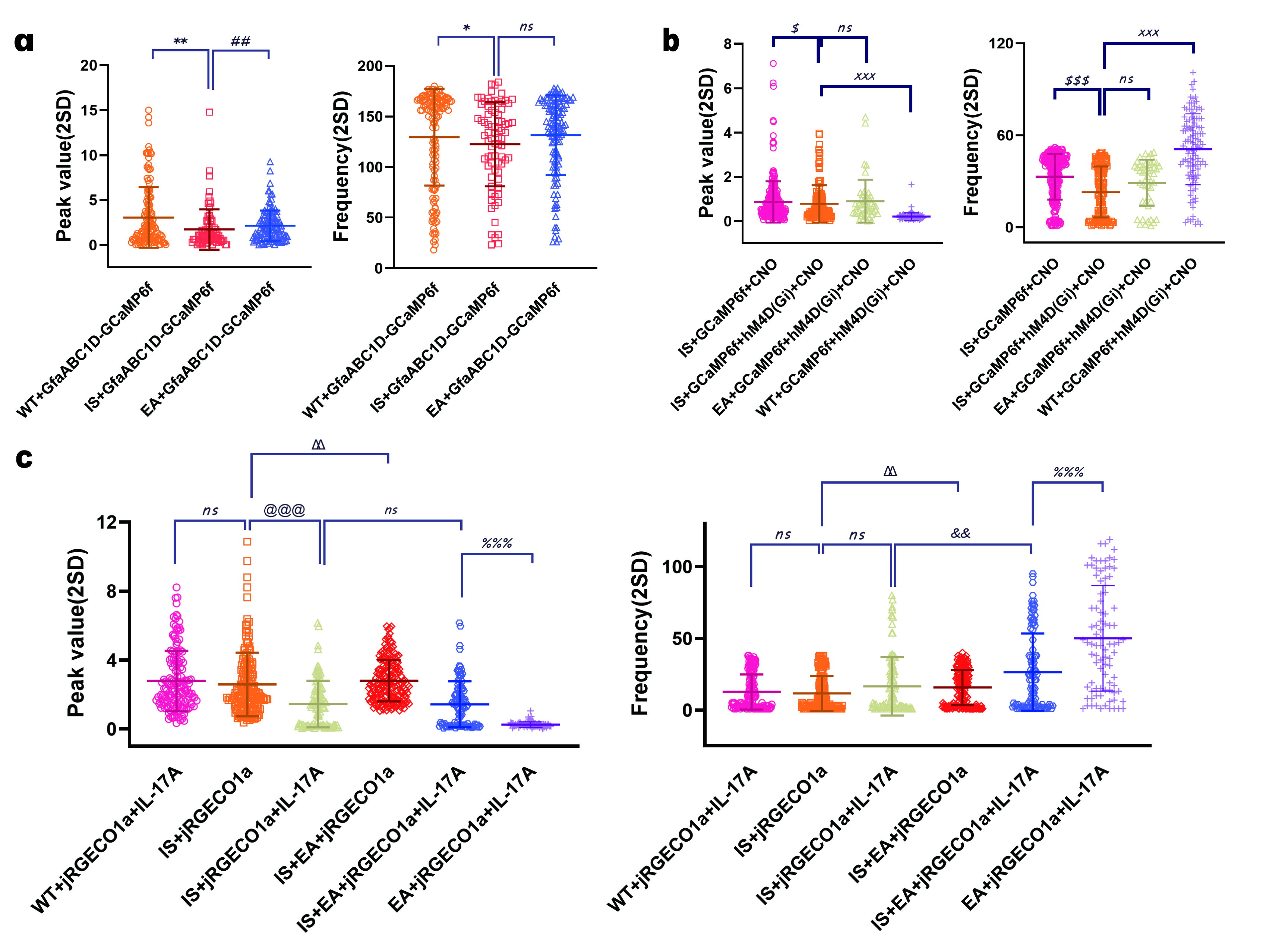

Supplement: Supplementary file 7 — Additional file7: Extended Data Fig. 3. a Comparative filtered analysis of Peak Value, and Frequency across WT+GfaABC1D-GCaMP6f, IS+GfaABC1D-GCaMP6f, and EA+GfaABC1D-GCaMP6f groups (n = 78–132). b Comparative filtered analysis of Peak Value, Frequency in IS + GCaMP6f + CNO, IS+GCaMP6f + hM4D(Gi) + CNO, EA+GCaMP6f+hM4D(Gi)+CNO groups, and WT+GCaMP6f+hM4D(Gi)+CNO (n = 47–264). c Comparative filtered analysis of Peak Value, Frequency in WT+jRGECO1a+IL-17A, IS+jRGECO1a, IS+jRGECO1a+IL-17A, IS+EA+jRGECO1a, IS+EA+jRGECO1a+IL-17A and EA+jRGECO1a+IL-17A groups (n = 89–189). Results are expressed as the mean ± SD, *p < 0.05, **p < 0.01, *** p < 0.001 vs. WT+GfaABC1D-GCaMP6f; ## p < 0.01 vs. IS+GfaABC1D-GCaMP6f; $$$ p < 0.001 vs. IS+GCaMP6f+CNO; ××× p < 0.001 vs. IS+GCaMP6f+hM4D(Gi)+CNO; &&& p < 0.001 vs. IS+jRGECO1a+IL-17A; @@@ p < 0.001 vs. IS+jRGECO1a; ∆∆ p < 0.01 vs. IS+EA+jRGECO1a; %%% p < 0.001 vs. EA+jRGECO1a+IL-17A. [file 13020_2026_1446_MOESM7_ESM.tif]
